# A Reproducible Multicentre MRI Radiomics Workflow for Pancreatic Cyst Risk Stratification Using Paired T1- and T2-Weighted Imaging

**DOI:** 10.3390/tomography12070100

**Published:** 2026-07-01

**Authors:** George Sgourakis

**Affiliations:** 1 HepatoPancreaticBiliary Unit, East Lancashire Hospitals NHS Trust, Blackburn BB2 3HH, UK; georgios.sgourakis@elht.nhs.uk; 2 School of Medicine, University of Lancashire, Preston PR1 2HE, UK

**Keywords:** pancreatic cyst, intraductal papillary mucinous neoplasm, magnetic resonance imaging, radiomics, multicentre study, machine learning, PyRadiomics, reproducibility

## Abstract

Purpose: To develop and technically validate a reproducible multicentre MRI radiomics workflow for pancreatic cyst risk stratification using paired T1- and T2-weighted imaging from public datasets. Methods: Public datasets were screened and Cyst-X was selected as the primary cohort because it contained pancreatic MRI, risk labels, masks and metadata. A linked Cyst-X subset was enriched with metadata, filtered to an exact paired T1/T2 cohort, and processed through image–mask quality control, 1.0 mm isotropic resampling, intensity normalisation, PyRadiomics feature extraction, feature reduction and patient-level centre-held-out validation. The revised modelling strategy used a T2 + clinical all-patient primary analysis (n = 409) and a complete-case paired T1/T2 sensitivity analysis (n = 299). Results: The final cohort comprised 409 patients and 818 image-level rows across EMC, IU, MCF and NYU. All 818 image–mask pairs passed post-preprocessing QC. T2 radiomics were complete for all 409 patients; however, 110 T1 feature sets were missing, all from MCF. In the all-patient T2 + clinical model comparison, logistic regression achieved the highest macro-AUC (0.737). The T2 + clinical random forest comparator achieved macro-AUC 0.716 (95% CI 0.678–0.755), accuracy 0.545 (95% CI 0.496–0.592) and macro-F1 0.530 (95% CI 0.481–0.577). The paired T1/T2 complete-case random forest sensitivity model achieved macro-AUC 0.735 (95% CI 0.691–0.777), accuracy 0.575 (95% CI 0.520–0.632) and macro-F1 0.554 (95% CI 0.494–0.605). Conclusion: This study demonstrates the feasibility of constructing a reproducible public data MRI radiomics workflow for pancreatic cyst risk stratification. Model performance was modest, and independent external validation is required before clinical application.

## Simple Summary

Pancreatic cysts are commonly found on MRI scans, but it can be difficult to identify which cysts are harmless and which may carry a higher risk of malignant transformation. This study tested whether public multicentre pancreatic MRI data could be converted into a reproducible radiomics workflow. Radiomics means extracting quantitative measurements from medical images, including lesion shape, signal intensity and texture. The workflow linked images, masks and labels, checked image–mask geometry, standardised preprocessing, extracted radiomics features and evaluated machine learning models while holding out each centre in turn. The revised analysis shows that T2-weighted MRI radiomics combined with age and sex provides modest discrimination for three-class pancreatic cyst risk stratification, while paired T1/T2 modelling was limited by incomplete T1 extraction in one centre. The study should therefore be interpreted as a technical feasibility and reproducibility study, not as a clinical decision-support tool.

## 1. Introduction

Pancreatic cystic lesions are increasingly identified on cross-sectional imaging. Risk stratification remains clinically challenging because conventional assessment relies on macroscopic features such as cyst size, duct calibre, mural nodules, interval change and symptoms, while many lesions remain indeterminate after routine imaging review [1,2]. Quantitative imaging may complement conventional interpretation by converting MRI data into structured descriptors of intensity, shape and texture [3,4].

Radiomics studies in pancreatic cystic lesions and intraductal papillary mucinous neoplasms have reported encouraging diagnostic performance, but translation remains limited by heterogeneous datasets, variable segmentation quality, inconsistent preprocessing, high-dimensional feature spaces, small sample sizes and inadequate external validation [5,6,7,8,9,10]. These limitations are especially relevant in public multicentre repositories, where image files, masks, labels and metadata may not be immediately analysis-ready.

The present study was designed as a technical feasibility and reproducibility study rather than a definitive clinical-effectiveness trial. The objective was to construct a transparent end-to-end MRI radiomics workflow for pancreatic cyst risk stratification using a public Cyst-X-derived paired T1/T2 cohort. The revised manuscript explicitly documents feature availability, radiomics extraction failures, feature reduction, patient-level validation, confidence intervals and the limits of centre-held-out internal validation. Supporting dataset, label, configuration, feature reduction and extended model-output details are provided in Tables S1–S13.

## 2. Materials and Methods

### 2.1. Study Design, Datasets and Clinical Labels

**Figure 1:**
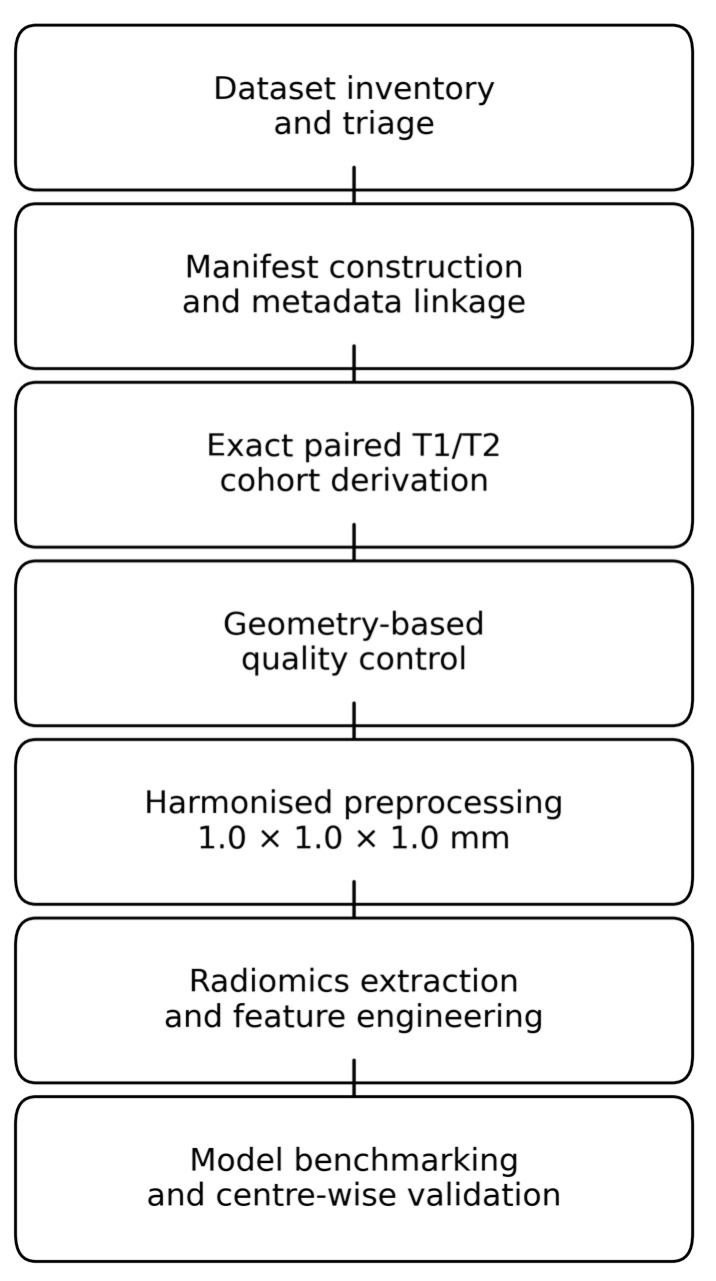
End-to-end analytic workflow from dataset inventory to centre-held-out validation.

**Figure 2:**
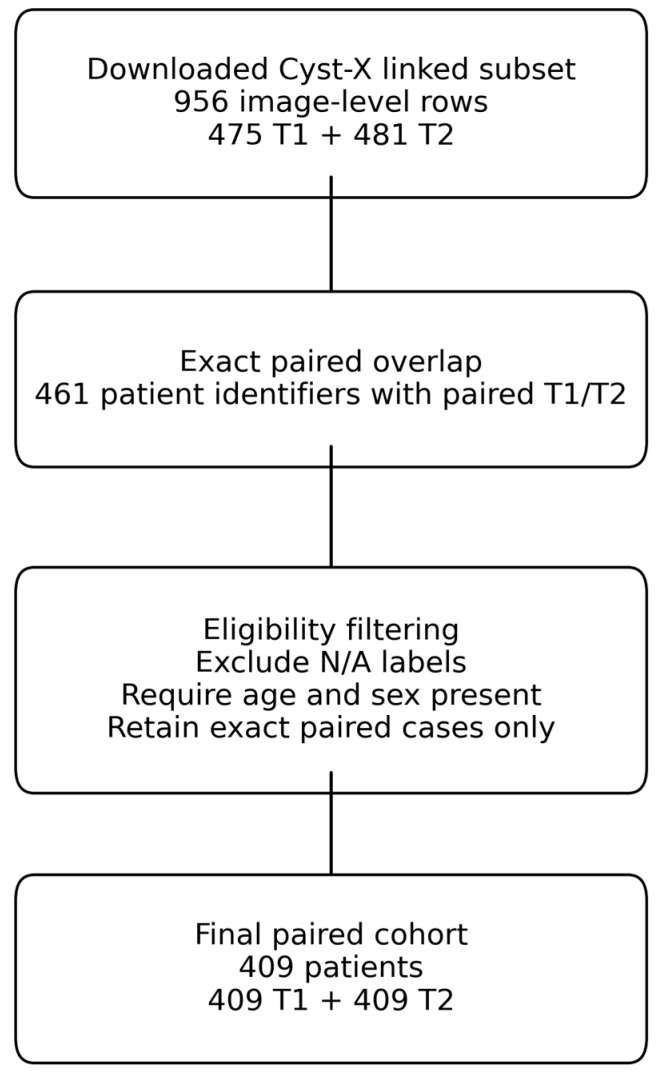
Cohort derivation from the linked public Cyst-X subset to the final paired analysis cohort.

**Figure 3:**
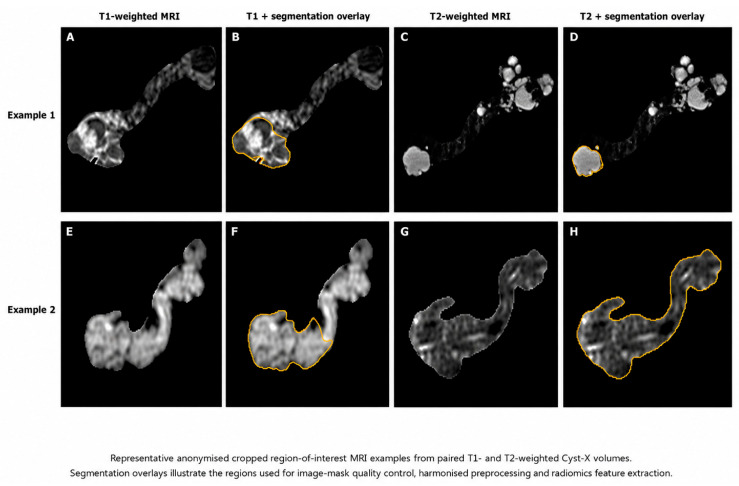
Representative paired Cyst-X MRI inputs with segmentation overlays (**A**–**H**). Representative anonymised cropped region-of-interest MRI examples generated from paired T1- and T2-weighted MRI volumes in the publicly available Cyst-X cohort. Panels (**A**–**D**) show Example 1 and panels (**E**–**H**) show Example 2. For each example, the columns show the T1-weighted MRI, T1 image with segmentation overlay, T2-weighted MRI and T2 image with segmentation overlay, respectively. Segmentation contours are overlaid to illustrate the regions used for image–mask quality control, harmonised preprocessing and radiomics feature extraction. Examples are shown for illustrative purposes only and were not selected on the basis of clinical label or model performance.

This was a retrospective multicentre methodological radiomics study using publicly available, de-identified pancreatic MRI datasets. Cyst-X was used as the primary analytic cohort because it provided pancreatic MRI, risk labels, masks and metadata [11]. PanSegData/PANSegNet and PANTHER (Figure 1 and Figure 2) were used as supporting resources for segmentation/preprocessing structure and format-resilience assessment [12,13]. Detailed dataset roles and clinical label definitions are provided in Tables S1 and S2. The Cyst-X risk labels were analysed as three dataset-defined categories: label 0, no-risk/control or non-high-risk comparator; label 1, low-risk cyst/IPMN; and label 2, high-risk cyst/IPMN. These labels were not re-adjudicated by an independent pathology or radiology panel in the present study. Representative paired T1- and T2-weighted Cyst-X MRI examples with segmentation overlays are shown in Figure 3 to illustrate the image-level inputs and corresponding regions of interest used for image–mask quality control, harmonised preprocessing and radiomics feature extraction.

### 2.2. Cohort Construction and Patient-Level Grouping

The initial linked downloaded Cyst-X subset contained 956 image-level rows: 475 T1-weighted and 481 T2-weighted studies. The manifest was enriched with age, sex, risk label linkage and exact T1/T2 pairing. Exact overlap identified 461 paired patient identifiers. After excluding N/A labels and cases with incomplete core metadata, the final paired cohort comprised 409 patients with 409 T1 and 409 T2 image-level rows. All downstream splitting, feature selection and validation were performed at patient level, with T1 and T2 data from the same patient kept together to avoid leakage. Detailed cohort derivation is provided in Table S3 and summarised in Table 1.

### 2.3. Image–Mask Quality Control and Preprocessing

An initial 40-row QC sample was performed across centres and modalities, followed by full-cohort QC of 818 image-level rows. QC evaluated image–mask dimension agreement, spacing agreement, mask datatype, non-zero mask voxel count and processing errors. Full images and their corresponding masks were used as the primary source. Images were standardised in orientation and resampled to 1.0 × 1.0 × 1.0 mm isotropic spacing. Linear interpolation was used for continuous MRI intensities, and nearest-neighbour interpolation was used for masks to preserve label integrity. After resampling, masks were enforced as integer labels and checked for non-zero voxels. Detailed QC and PyRadiomics configuration parameters are provided in Tables S4 and S5, with additional visual QC summaries in Figures S1–S3.

MRI intensity normalisation was performed before radiomics extraction using percentile clipping followed by z-score normalisation. In mathematical terms, voxel intensities were clipped to predefined lower and upper percentile bounds, and the normalised intensity was computed as z = (x − mu)/sigma, where mu and sigma were estimated from the non-background image region. Original spacing and dimension metadata were retained for traceability.

### 2.4. Inter-Sequence Registration and Multimodal Fusion

No additional rigid, affine or deformable T1-to-T2 co-registration was performed before radiomics extraction. Instead, each modality was analysed using its corresponding image and segmentation mask after image–mask geometry checks and harmonised preprocessing. The T1 and T2 features were fused at the patient level rather than interpreted as voxel-wise co-registered inter-sequence features. This distinction is important because T1- and T2-weighted MRI sequences may be acquired at different time points and may be affected by motion, respiratory variation or bowel peristalsis.

### 2.5. Radiomics Extraction and Standardisation

Radiomics extraction was performed using PyRadiomics version 3.1.0 through a scripted Python 3.13.5 workflow [4]. The extraction script recorded patient identifier, centre, modality, image path, mask path, risk label, age, sex, QC pass status, extraction status and extraction errors for every patient-modality row. Diagnostic PyRadiomics fields were excluded from the modelling matrix. The YAML extraction configuration used bin width 25, label 1, geometry tolerance 0.01, mask correction enabled, original image type and the following feature classes: first-order, shape, GLCM, GLRLM, GLSZM, GLDM and NGTDM. PyRadiomics resampling and normalisation were disabled in the YAML because images had already been resampled and normalised during preprocessing. Detailed configuration and extraction completeness are provided in Tables S4–S6, and the distribution of raw feature families is shown in Figure S4.

### 2.6. Feature Preprocessing, Feature Reduction and Leakage Prevention

Because the raw radiomics matrix was high-dimensional relative to the number of patients, feature reduction was performed before model training. All feature preprocessing was performed within the training data only for each centre-held-out split. Missing values were imputed from the training data where applicable, near-zero variance features were removed, and highly correlated features were pruned using a pairwise correlation threshold of |rho| > 0.95. For correlated feature pairs, the retained feature was selected using lower missingness and stronger training-set association with the outcome. The held-out centre was not used during imputation, scaling, correlation filtering or feature selection.

The primary analysis used the complete all-patient T2 + clinical feature set (n = 409), because T2 radiomics were available for all patients. Age and sex were included as available clinical metadata. A complete-case paired T1/T2 sensitivity analysis was also performed in patients with both T1 and T2 radiomics available (n = 299). No imputation of entirely missing T1 radiomics feature sets was used for the paired complete-case analysis. Detailed feature availability, feature reduction and feature importance outputs are provided in Tables S6–S9.

### 2.7. Model Development, Centre-Held-Out Validation and Statistical Reporting

The revised modelling analysis compared an age/sex clinical baseline with T2 + clinical logistic regression, support vector machine, random forest and gradient boosting models. A paired T1/T2 complete-case random forest was retained as a sensitivity analysis. Class imbalance was addressed using class weighting where supported by the classifier. Random forest models used inverse-frequency class weighting, where w_k = N/(K n_k), N is the number of training samples, K is the number of classes and n_k is the number of training samples in class k. Tree splitting used the Gini impurity criterion, G = 1 − sum(p_k^2^).

Model validation was performed using leave-one-centre-out (LOCO) validation. For each iteration, three centres were used for training and the fourth centre was held out for testing. LOCO validation was treated as centre-held-out internal validation, not true external validation. Performance was reported using macro one-versus-rest AUC, weighted one-versus-rest AUC, accuracy, balanced accuracy, macro-F1, weighted-F1 and multiclass Brier score. Confidence intervals were estimated by patient-level bootstrap resampling of the LOCO predictions. Extended model comparison and confidence interval outputs are provided in Tables S10–S12.

## 3. Results

### 3.1. Cohort Readiness, Preprocessing and Radiomics Completeness

The final paired cohort comprised 409 patients with paired T1/T2 imaging, corresponding to 818 image-level rows across four centres. The final label distribution was 0:110, 1:186 and 2:113. The centre distribution was EMC 70, IU 62, MCF 130 and NYU 147. All 409 patients passed post-preprocessing QC, and the full 818-row preprocessing run completed without errors. Radiomics extraction produced complete T2 radiomics for all 409 patients. In contrast, 110 T1 feature sets were missing, all from MCF. Therefore, the all-patient model was based on T2 radiomics plus age and sex, while paired T1/T2 modelling was retained only as a complete-case sensitivity analysis in 299 patients. Detailed rows by centre, modality and label are provided in Table S13.

### 3.2. Feature Reduction and Retained Features

The primary T2 + clinical analysis retained 68 predictors after training-only variance filtering and correlation pruning. The complete-case paired T1/T2 sensitivity analysis retained 122 predictors. The highest-ranking T2 + clinical random forest features included age and multiple T2 first-order intensity dispersion measures, including mean absolute deviation, interquartile range, root mean squared intensity and energy. The paired complete-case sensitivity model retained several T1 texture and first-order features in addition to T2 intensity features and age. Full feature importance outputs are provided in Tables S8 and S9.

### 3.3. Model Performance

Table 2 summarises the revised available-metadata model comparison. The age/sex clinical baseline had macro-AUC 0.665. Among all-patient T2 + clinical models, logistic regression achieved the highest macro-AUC (0.737), followed by random forest (0.716), gradient boosting (0.710) and support vector machine (0.690). Although the random forest model achieved slightly higher accuracy than logistic regression, logistic regression provided the strongest macro-AUC discrimination. This suggests that, after feature filtering and correlation pruning, much of the retained predictive signal may be approximately additive or linearly separable, while more flexible nonlinear models may be more susceptible to centre-specific overfitting in this modestly sized multicentre cohort. The paired T1/T2 complete-case random forest sensitivity model had macro-AUC 0.735 in 299 patients. Because all results were based on inherited public labels and centre-held-out internal validation, the discrimination should be interpreted as modest technical feasibility rather than clinical readiness.

### 3.4. Centre-Held-Out Random Forest Performance

Table 3 reports the centre-held-out random forest comparator and sensitivity analyses. The T2 + clinical random forest comparator achieved overall macro-AUC 0.716 (95% CI 0.678–0.755), accuracy 0.545 (95% CI 0.496–0.592), balanced accuracy 0.519 (95% CI 0.469–0.568) and macro-F1 0.530 (95% CI 0.481–0.577). The paired T1/T2 complete-case random forest sensitivity analysis achieved overall macro-AUC 0.735 (95% CI 0.691–0.777), accuracy 0.575 (95% CI 0.520–0.632), balanced accuracy 0.548 (95% CI 0.491–0.606) and macro-F1 0.554 (95% CI 0.494–0.605). Centre-specific performance varied, especially in the smaller IU and complete-case MCF subsets, emphasising the need for larger external validation cohorts.

## 4. Discussion

This study should be read primarily as a technical reproducibility and feasibility paper. Its main contribution is not that it delivers a clinically ready pancreatic cyst classifier, but that it documents the practical steps required to convert a public multicentre pancreatic MRI resource into an auditable radiomics workflow. The workflow begins with dataset inventory and metadata linkage, proceeds through exact T1/T2 pairing, patient-level cohort derivation, image–mask quality control, spatial harmonisation, intensity normalisation and PyRadiomics extraction, and then moves to feature reduction and centre-held-out model evaluation. This end-to-end transparency is important because many radiomics studies report headline discrimination metrics while giving insufficient detail about how heterogeneous images, masks, preprocessing settings, missing features and validation partitions were handled.

The clinical motivation remains strong. Pancreatic cystic neoplasms, particularly IPMN, create a recurrent clinical dilemma: unnecessary surgery exposes patients to morbidity, while missed high-grade dysplasia or invasive carcinoma carries major consequences. International and European guidelines provide structured risk frameworks based on clinical and morphological variables [1,2]. However, guideline-based decision-making still leaves many patients in indeterminate surveillance pathways. Radiomics is attractive because it may quantify intensity heterogeneity, shape irregularity and texture patterns that are difficult to express in conventional reports. In this study, the highest-ranking features in the primary T2 + clinical model were age and T2 first-order intensity dispersion features, suggesting that signal-distribution heterogeneity, rather than simple binary morphology alone, contributed to discrimination. This is biologically plausible for cystic lesions, where mural complexity, internal debris, mucin, wall irregularity and adjacent parenchymal changes may alter intensity distributions.

The modelling results should be interpreted with caution. In the all-patient T2 + clinical comparison, logistic regression achieved the highest macro-AUC (0.737), whereas the T2 + clinical random forest comparator achieved macro-AUC 0.716 and accuracy 0.545 in centre-held-out validation. These values are modest, and they are lower than the most optimistic performance estimates sometimes reported in radiomics and deep-learning studies. That is not necessarily a negative finding. A centre-held-out validation design is deliberately more conservative than random train–test splitting, because it tests whether the model can transfer across institutional acquisition differences. In this context, modest performance may be a more realistic estimate of what can be expected when a model is applied to data from an unseen centre. The paired T1/T2 complete-case random forest sensitivity analysis produced a slightly higher macro-AUC of 0.735, but this analysis included only 299 patients and was affected by severe centre-specific missingness, particularly the limited complete-case MCF subset. The apparent improvement with paired T1/T2 features should therefore be interpreted as hypothesis-generating rather than definitive.

The observation that logistic regression outperformed random forest by macro-AUC in the all-patient T2 + clinical analysis is methodologically informative. After near-zero variance filtering and correlation-based pruning, the retained feature space may have contained predominantly additive signal from age and T2 intensity distribution features. In a modestly sized three-class cohort with centre-held-out validation, a simpler regularised linear model may generalise better than a flexible ensemble model that can capture centre-specific thresholds or acquisition-related artefacts. Random forest nevertheless remained useful as a nonlinear comparator, for feature importance exploration, and for the paired T1/T2 complete-case sensitivity analysis. The results therefore suggest that model simplicity may be advantageous in early multicentre radiomics feasibility work, particularly when sample size, centre imbalance and feature missingness constrain model complexity.

The revised analysis also clarifies an important methodological lesson: preprocessing success does not guarantee radiomics extraction completeness. All 818 image–mask pairs passed post-preprocessing QC, yet 110 T1 radiomics feature sets were missing at the extraction stage. This is exactly the kind of failure mode that should be visible in reproducible radiomics work. Reporting the T1 extraction failure, rather than hiding it behind a pooled performance number, improves the credibility of the workflow and directly addresses reproducibility concerns. The consequence is that T2 radiomics became the appropriate all-patient primary analysis, while paired T1/T2 modelling became a complete-case sensitivity analysis. This distinction is important for future users of the workflow, because missingness that is concentrated within a centre can bias model development and centre-held-out validation if it is handled as ordinary random missingness.

The study also sits within a broader methodological context. The Image Biomarker Standardisation Initiative emphasises that radiomics features require standardised definitions and reporting of upstream image processing steps [5]. PyRadiomics provides a widely used implementation, but reproducibility still depends on reporting voxel spacing, interpolation, intensity normalisation, discretisation bin width, enabled image types, feature classes, mask labels and software versioning [4,5]. The revised manuscript therefore reports the preprocessing specification and PyRadiomics YAML configuration in the Supplementary Material. This is particularly important for MRI, where intensities are not inherently standardised across scanners or sites. Unlike CT Hounsfield units, MRI signal intensity is affected by scanner vendor, sequence parameters, coil configuration, field strength, patient habitus and reconstruction. The use of percentile clipping and within-image z-score normalisation is pragmatic, but it does not eliminate all centre effects or guarantee feature stability. Future work should include test–retest data, repeated segmentation, perturbation analysis and phantom or benchmark feature checks where feasible.

The comparison with existing AI and radiomics work is necessarily nuanced. Cyst-X and related studies demonstrate the promise of multicentre MRI-based IPMN risk classification and federated learning [10,11]. Other pancreatic cyst radiomics studies have used CT or different classification endpoints [8,9]. These studies are useful context, but they are not directly interchangeable with the present analysis because modality, labels, inclusion criteria, validation design and available clinical covariates differ. The present manuscript is deliberately narrower: it asks whether a public MRI dataset can be converted into a traceable radiomics workflow with honest documentation of feature completeness and centre-held-out performance. Its value is therefore more methodological than competitive. It provides a baseline that future work can extend with stronger segmentation QC, full clinical-imaging predictors, external test cohorts and prospective validation.

The request for comparison with clinical models is also important. The available reconstructed dataset permitted an age/sex clinical baseline, but it did not include the complete set of clinical and imaging predictors required for a guideline-based model, such as cyst size, main pancreatic duct diameter, mural nodule status, jaundice, pancreatitis, CA19-9, cyst growth rate, cytology or molecular markers. Consequently, the reported clinical comparator is an available-metadata baseline, not a formal Fukuoka, European or Kyoto guideline model. This distinction should prevent overinterpretation. The appropriate next study should test incremental value: guideline variables alone, radiomics alone, and combined guideline–radiomics models, ideally with calibration, decision-curve analysis and predefined thresholds for high-risk disease.

From a reporting perspective, the manuscript has been aligned more closely with contemporary expectations for medical imaging AI and prediction modelling. The study was reported with reference to the CLAIM and TRIPOD+AI reporting guidelines, which emphasise transparent reporting of data sources, patient-level grouping, model development, validation design, missing data handling, calibration and limitations [14,15,16]. This study still falls short of a full clinical prediction model because it lacks independent external validation, prospective data and comprehensive clinical predictors. However, the study improves the reporting of methodological elements that are often under-specified in feasibility radiomics papers, including patient-level splitting, feature reduction within training folds, centre-held-out testing, bootstrap confidence intervals and explicit declaration of technical extraction failures.

Clinical translation would require several additional steps. First, the pipeline should be rerun on an independent external cohort from a hospital not represented in the current four-centre subset. Second, T1 extraction failures should be resolved or the workflow should predefine a T2-first model if T2 proves more robust across centres. Third, segmentation variability should be quantified using manual and semi-automated masks from multiple observers. Fourth, radiomics should be compared with guideline-based predictors and radiologist interpretation using calibrated probabilities and clinically relevant thresholds. Finally, data and code should be deposited with enough detail to allow an independent group to reproduce the cohort, preprocessing, extraction and modelling outputs. Until these steps are completed, the workflow should be considered a research platform, not a clinical decision-support system.

### Limitations

This study has several important limitations. First, it is retrospective and relies on public datasets. Although the data are multicentre in origin, they are curated public data and may not fully represent routine clinical MRI variability, including motion artefact, scanner vendor variation, inconsistent reporting and incomplete clinical covariates. Second, clinical labels were inherited from the public dataset and were not independently re-adjudicated. Third, full guideline-based predictors such as cyst size, main pancreatic duct diameter, mural nodules, CA19-9, pancreatitis history and cyst-fluid cytology were not available in the reconstructed radiomics table. The clinical baseline is therefore an age/sex available-metadata baseline rather than a Fukuoka, European or Kyoto guideline model.

Fourth, no additional rigid, affine or deformable inter-sequence T1-to-T2 co-registration was performed. The analysis should therefore be understood as patient-level multimodal fusion rather than voxel-wise registered T1/T2 radiomics. Fifth, T1 radiomics extraction failed in 110 MCF cases, leaving only 299 complete paired T1/T2 cases and only 20 complete MCF cases for paired complete-case LOCO testing. Sixth, feature reproducibility was not assessed using scan–rescan MRI or repeated segmentations. Seventh, model calibration was assessed only through available prediction summaries and was not supported by prospective recalibration. Finally, the study should not be interpreted as clinical readiness or universal multicentre robustness. It is a technical feasibility proof-of-concept requiring independent external validation and prospective testing.

## 5. Conclusions

In this technical feasibility study, a public multicentre pancreatic MRI dataset was converted into a reproducible, centre-aware radiomics workflow using paired T1- and T2-weighted imaging. The principal contribution is methodological: a documented pipeline for cohort derivation, image–mask quality control, harmonised preprocessing, radiomics extraction, feature reduction and centre-held-out validation. The revised analysis shows that T2-derived radiomics combined with available clinical metadata provides modest discrimination for three-class pancreatic cyst risk stratification, with paired T1/T2 complete-case modelling retained as a sensitivity analysis. These findings support further methodological development but should not be interpreted as clinical readiness. Independent external validation, prospective testing and comparison with full guideline-based clinical-imaging predictors are required before clinical deployment.

## Figures and Tables

**Table 1:** Core cohort, preprocessing and radiomics completeness summary.

Domain	Manuscript Summary
Final analysis cohort	409 paired patients; 409 T1 and 409 T2 image-level rows
Centres	EMC 70; IU 62; MCF 130; NYU 147
Risk label distribution	0:110; 1:186; 2:113
Post-preprocessing QC	818/818 rows OK; 0 errors; 818/818 image–mask dimension matches; 818/818 spacing matches; 0 empty masks; 0 NaN/Inf rows
Radiomics features	107 T1 and 107 T2 candidate radiomics features in the reconstructed patient-level table
Radiomics completeness	T2 complete in 409/409 patients; T1 missing in 110/409 patients, all from MCF; complete paired T1/T2 radiomics available in 299/409 patients
Primary modelling analysis	T2 radiomics + age + sex, n = 409
Sensitivity modelling analysis	Complete-case paired T1/T2 radiomics + age + sex, n = 299

Detailed dataset, label, QC, PyRadiomics and feature availability tables are provided in Tables S1–S6. QC, quality control; EMC, Erasmus Medical Center; IU, Istanbul University Faculty of Medicine; MCF, Mayo Clinic Florida; NYU, New York University Langone Health.

**Table 2:** Revised available-metadata model comparison.

Model	n	Macro-AUC	Weighted AUC	Accuracy	Balanced Accuracy	Macro-F1
T2 + clinical Logistic regression	409	0.737	0.713	0.531	0.540	0.536
T2 + clinical Support vector machine	409	0.690	0.661	0.553	0.548	0.551
T2 + clinical random forest	409	0.716	0.692	0.545	0.519	0.530
T2 + clinical gradient boosting	409	0.710	0.694	0.560	0.536	0.546
Age + sex clinical baseline	409	0.665	0.651	0.469	0.492	0.478
Paired T1/T2 complete-case RF	299	0.735	0.704	0.575	0.543	0.554

Full model comparison and bootstrap confidence intervals are provided in Tables S10 and S11. AUC = area under the receiver operating characteristic curve; RF = random forest.

**Table 3:** Random forest comparator and sensitivity LOCO performance with bootstrap 95% confidence intervals.

Analysis	n	Macro-AUC	Accuracy	Balanced Accuracy	Macro-F1	Multiclass Brier
T2 + clinical RF LOCO comparator	409	0.716 (0.678–0.755)	0.545 (0.496–0.592)	0.519 (0.469–0.568)	0.530 (0.481–0.577)	0.567 (0.529–0.605)
Sensitivity paired T1/T2 complete-case RF LOCO	299	0.735 (0.691–0.777)	0.575 (0.520–0.632)	0.543 (0.486–0.596)	0.554 (0.494–0.605)	0.543 (0.497–0.594)

Detailed centre-specific metrics are provided in Table S12. Brier = multiclass Brier score; LOCO = leave-one-centre-out.

## Data Availability

The study used publicly available datasets, including Cyst-X, PanSegData/PANSegNet and the PANTHER public training dataset [11,12,13]. Derived cohort summaries, QC summaries, radiomics extraction summaries and revised model-output tables are provided as Supplementary Files. The preprocessing specification, PyRadiomics YAML configuration, extraction script and revised reanalysis tables are provided as Supplementary Material. The corresponding author will deposit the final code package in a public repository such as GitHub after publication or upon journal request.

## Supplementary Materials

The following supporting information can be downloaded at: https://www.mdpi.com/article/10.3390/tomography12070100/s1, Table S1. Public datasets screened and their role in the workflow; Table S2. Clinical interpretation of Cyst-X risk labels used in this study; Table S3. Detailed cohort derivation and final cohort composition; Table S4. Preprocessing and PyRadiomics configuration; Table S5. Detailed QC, preprocessing and radiomics-extraction summary; Table S6. Feature availability and revised analysis sets; Table S7. Feature-reduction strategy used in the revised modelling analyses; Table S8. Full retained-feature importance table for the T2 + clinical random forest comparator model; Table S9. Full retained-feature importance table for the paired T1/T2 complete-case random forest sensitivity model; Table S10. Full revised available-metadata model comparison output; Table S11. Random forest comparator and sensitivity LOCO bootstrap confidence intervals; Table S12. Random forest comparator centre-held-out metrics with bootstrap AUC confidence intervals; Table S13. Radiomics rows by centre, modality and risk label; Figure S1. Full-cohort mask voxel distribution by centre and modality; Figure S2. Distribution of pre-normalisation mean intensities by modality; Figure S3. Full-cohort image–mask QC summary; Figure S4. Radiomics feature families in the raw extraction file; Supplementary code/configuration files: The supplementary code/configuration files include the PyRadiomics YAML parameter file and the radiomics ex-traction script used to support reproducibility of the preprocessing and feature-extraction workflow.
