# Supplementary material for: A Reproducible Multicentre MRI Radiomics Workflow for Pancreatic Cyst Risk Stratification Using Paired T1- and T2-Weighted Imaging
Source: Tomography. 2026 Jul 1;12(7):100. doi: 10.3390/tomography12070100 (PMC13417891; doi:10.3390/tomography12070100)
Supplement: Supplementary file 1 [file tomography-12-00100-s001.zip › tomography-4354956-supplementary.pdf]

Supplementary Tables

A Reproducible Multi-Centre MRI Radiomics Workflow for Pancreatic Cyst Risk Stratification Using Paired T1- and T2-Weighted Imaging

Table S1. Public datasets screened and their role in the workflow

Dataset	Imaging content	Labels/masks	Format	Role in study
Cyst-X	Paired T1/T2 pancreatic MRI	Risk labels, lesion/pancreas masks and metadata	NIfTI	Primary analytic cohort
PanSegData/PANSegNet	T1/T2 pancreatic MRI and CT with structured labels	Segmentation labels	nnUNet-style	Segmentation and preprocessing support
PANTHER public training dataset	Diagnostic MRI and MR-linac MRI	Partially labelled cases and metadata	.mha and related metadata	Format-resilience stress testing

Table S2. Clinical interpretation of Cyst-X risk labels used in this study

Label	Clinical interpretation	Use in this study
0	No-risk/control or non-high-risk comparator category	Class 0 in the three-class classification task
1	Low-risk cyst/IPMN category	Class 1 in the three-class classification task
2	High-risk cyst/IPMN category	Class 2 in the three-class classification task

Table S3. Detailed cohort derivation and final cohort composition

Cohort metric	Value
Initial linked downloaded Cyst-X subset	956 image-level rows
T1 rows in linked subset	475
T2 rows in linked subset	481
Exact paired T1/T2 overlap	461 patient identifiers
Final paired analysis cohort after excluding N/A labels and incomplete metadata	409 patients; 409 T1 and 409 T2 image-level rows
Risk-label distribution	0: 110; 1: 186; 2: 113
Centre distribution	EMC: 70; IU: 62; MCF: 130; NYU: 147
Sex distribution	Female: 226; Male: 183

Table S4. Preprocessing and PyRadiomics configuration

Parameter	Value
Source images	Full images and masks were treated as the primary analysis source; ROI-cropped files were not used as the definitive modelling source
Target spacing	1.0 mm × 1.0 mm × 1.0 mm isotropic voxel spacing.
Image interpolation	Linear interpolation for continuous MRI intensities during preprocessing
Mask interpolation	Nearest-neighbour interpolation for binary/label masks
Intensity normalisation	Percentile clipping followed by within-image z-score normalisation of the non-background region before radiomics extraction
Radiomics engine	PyRadiomics version 3.1.0, run through a scripted Python 3.13.5 workflow using the supplied YAML extraction file and extraction script.
PyRadiomics resampledPixelSpacing	null, because images were already resampled during preprocessing
PyRadiomics normalise flag	false, because normalisation was performed before extraction
Bin width	25
Image type	Original only
Feature classes	firstorder, shape, GLCM, GLRLM, GLSZM, GLDM and NGTDM
Mask label	1
Geometry tolerance	0.01
Mask correction	correctMask: true

Table S5. Detailed QC, preprocessing and radiomics-extraction summary

Workflow stage	Summary
Initial QC sample	40 rows assessed; 40/40 image-mask dimension matches; 40/40 spacing matches; 0 empty masks; 0 errors
Full paired-cohort QC before preprocessing	818 rows assessed; 818/818 dimension matches; 818/818 spacing matches; 0 empty masks; 0 errors
Full preprocessing QC	818/818 rows OK; 0 errors; 818/818 dimension matches; 818/818 spacing matches; 0 empty masks; 0 NaN/Inf rows
Radiomics raw output	818 rows; 409 unique patients; 2 modalities; 107 radiomics features per modality in patient-level reconstruction
Radiomics extraction completeness	708 complete image-level radiomics rows; 110 image-level rows with missing feature values; T2 complete for 409/409 patients; complete paired T1/T2 radiomics for 299/409 patients

Table S6. Feature availability and revised analysis sets

Analysis set	n	Feature availability	Role in revised manuscript
All-patient T2+clinical primary analysis	409	T2 radiomics available for all patients; age and sex available	Primary analysis because it uses the complete public cohort without imputing missing T1 feature sets
Paired T1/T2 complete-case sensitivity analysis	299	Both T1 and T2 radiomics available	Sensitivity analysis to test whether adding T1 features changes performance
Missing T1 feature sets	110	All from MCF T1 extraction rows	Handled transparently as a technical failure mode; no imputation of missing T1 radiomics was used for the complete-case analysis

Table S7. Feature-reduction strategy used in the revised modelling analyses

Analysis	Candidate predictors	Reduction strategy	Retained predictors
Primary T2+clinical	T2 radiomics + age + sex	Training-only variance filtering, median imputation where needed, correlation filtering at |rho| > 0.95, class-balanced model training	68 retained predictors
Paired complete-case sensitivity	T1 + T2 radiomics + age + sex	Complete-case filtering, training-only variance and correlation filtering, class-balanced random forest	122 retained predictors

Table S8. Full retained-feature importance table for the T2+clinical random forest comparator model

Feature	RF importance	Univariate F
age	0.073033	62.453
t2__original_firstorder_MeanAbsoluteDeviation	0.066954	56.995
t2__original_firstorder_InterquartileRange	0.053324	46.092
t2__original_firstorder_RootMeanSquared	0.039504	50.721
t2__original_firstorder_Energy	0.034855	30.286
t2__original_firstorder_90Percentile	0.029128	29.540
t2__original_firstorder_Kurtosis	0.020348	21.830
t2__original_glcm_JointEntropy	0.016855	1.353
t2__original_glrlm_RunEntropy	0.016812	18.462
t2__original_shape_Sphericity	0.016713	9.191
t2__original_glcm_Imc1	0.016657	9.733
t2__original_glcm_MCC	0.016438	7.376
t2__original_shape_SurfaceVolumeRatio	0.016041	12.840
t2__original_firstorder_Mean	0.015552	11.267
t2__original_glszm_SmallAreaLowGrayLevelEmphasis	0.015153	1.608
t2__original_glcm_Imc2	0.014722	4.513
t2__original_glszm_SmallAreaEmphasis	0.014673	0.792
t2__original_gldm_SmallDependenceHighGrayLevelEmphasis	0.014246	3.036
t2__original_firstorder_Minimum	0.013996	12.139
t2__original_glrlm_LowGrayLevelRunEmphasis	0.013937	4.750
t2__original_glrlm_ShortRunLowGrayLevelEmphasis	0.013795	2.814
t2__original_glszm_GrayLevelNonUniformityNormalized	0.013234	6.431
t2__original_firstorder_Skewness	0.012955	12.076
t2__original_shape_LeastAxisLength	0.012727	4.961
t2__original_firstorder_Maximum	0.012579	2.710
t2__original_firstorder_Range	0.012488	3.315
t2__original_shape_Maximum2DDiameterSlice	0.012377	2.089
t2__original_shape_Flatness	0.012031	7.246
t2__original_glcm_ClusterProminence	0.011974	2.022
t2__original_glszm_SmallAreaHighGrayLevelEmphasis	0.011929	3.768
t2__original_firstorder_10Percentile	0.011860	5.281
t2__original_shape_MinorAxisLength	0.011761	0.252
t2__original_glrlm_ShortRunHighGrayLevelEmphasis	0.011594	6.209
t2__original_glszm_HighGrayLevelZoneEmphasis	0.011580	1.881
t2__original_gldm_SmallDependenceLowGrayLevelEmphasis	0.011319	4.324
t2__original_glszm_ZoneVariance	0.011029	3.954
t2__original_glszm_SizeZoneNonUniformityNormalized	0.010840	0.747
t2__original_shape_MeshVolume	0.010838	6.268
t2__original_gldm_DependenceNonUniformity	0.010737	6.103
t2__original_glszm_SizeZoneNonUniformity	0.010713	1.529
t2__original_gldm_GrayLevelNonUniformity	0.010666	4.931
t2__original_glszm_ZoneEntropy	0.010023	3.304
t2__original_glszm_GrayLevelNonUniformity	0.010021	0.701
t2__original_glrlm_RunVariance	0.009981	5.047
t2__original_glszm_LargeAreaLowGrayLevelEmphasis	0.009590	0.763
t2__original_glcm_Idn	0.009576	1.836
t2__original_shape_MajorAxisLength	0.009481	1.170
t2__original_shape_Maximum3DDiameter	0.009476	0.332
t2__original_glszm_ZonePercentage	0.009461	1.062
t2__original_shape_Maximum2DDiameterRow	0.009351	1.286
t2__original_shape_Maximum2DDiameterColumn	0.009279	0.339
t2__original_glrlm_LongRunLowGrayLevelEmphasis	0.009267	0.673
t2__original_shape_SurfaceArea	0.009187	1.802
t2__original_glcm_ClusterTendency	0.009143	2.952
t2__original_gldm_LargeDependenceHighGrayLevelEmphasis	0.009093	4.694
t2__original_glszm_LargeAreaHighGrayLevelEmphasis	0.008987	5.165
t2__original_glcm_ClusterShade	0.008911	0.890
t2__original_shape_Elongation	0.008883	0.947
t2__original_ngtdm_Strength	0.008737	1.248
t2__original_glrlm_GrayLevelVariance	0.008723	0.554
t2__original_glrlm_ShortRunEmphasis	0.008441	1.722
t2__original_glrlm_LongRunHighGrayLevelEmphasis	0.008069	7.462
t2__original_glrlm_GrayLevelNonUniformity	0.007882	1.606
t2__original_glrlm_RunLengthNonUniformityNormalized	0.007611	3.524
t2__original_gldm_DependenceVariance	0.007457	1.868

t2__original_ngtdm_Busyness	0.007172	0.407
t2__original_gldm_SmallDependenceEmphasis	0.006805	3.937
gender_male	0.001425	3.296

Table S9. Full retained-feature importance table for the paired T1/T2 complete-case random forest sensitivity model

Feature	RF importance	Univariate F
t1__original_glcm_Imc2	0.047599	71.706
t2__original_firstorder_MeanAbsoluteDeviation	0.041506	45.425
age	0.033119	53.601
t1__original_firstorder_10Percentile	0.031269	71.831
t1__original_glrlm_GrayLevelVariance	0.027957	48.301
t1__original_glcm_Correlation	0.023858	31.312
t2__original_firstorder_InterquartileRange	0.022910	35.230
t1__original_glcm_Imc1	0.021919	50.702
t1__original_ngtdm_Strength	0.020243	9.237
t2__original_firstorder_90Percentile	0.020230	31.889
t2__original_firstorder_RootMeanSquared	0.020048	46.498
t2__original_firstorder_Energy	0.019542	27.491
t1__original_glrlm_ShortRunEmphasis	0.015298	31.557
t1__original_gldm_DependenceEntropy	0.014622	36.733
t1__original_firstorder_Mean	0.011917	37.576
t1__original_firstorder_Maximum	0.010897	14.014
t1__original_firstorder_Kurtosis	0.010805	22.692
t1__original_glrlm_ShortRunHighGrayLevelEmphasis	0.010369	25.997
t2__original_glcm_Imc1	0.009819	7.512
t2__original_shape_Sphericity	0.009666	5.520
t1__original_glszm_ZonePercentage	0.009554	15.629
t1__original_firstorder_Minimum	0.009352	30.922
t1__original_gldm_LargeDependenceLowGrayLevelEmphasis	0.009309	4.715
t1__original_firstorder_TotalEnergy	0.009180	18.351
t2__original_glrlm_RunVariance	0.008983	7.177
t1__original_gldm_DependenceVariance	0.008966	27.739
t1__original_glszm_ZoneEntropy	0.008851	42.427
t2__original_firstorder_Mean	0.008810	17.452
t2__original_shape_SurfaceVolumeRatio	0.008613	12.021
t1__original_firstorder_Range	0.008577	2.853
t2__original_gldm_GrayLevelNonUniformity	0.008521	9.642
t1__original_glrlm_RunLengthNonUniformityNormalized	0.008352	20.126
t1__original_firstorder_InterquartileRange	0.008299	11.476
t1__original_glszm_LargeAreaHighGrayLevelEmphasis	0.008205	20.354
t2__original_firstorder_Kurtosis	0.007980	18.273
t1__original_gldm_SmallDependenceHighGrayLevelEmphasis	0.007939	15.099
t2__original_gldm_DependenceEntropy	0.007865	3.612
t1__original_glrlm_ShortRunLowGrayLevelEmphasis	0.007808	17.984
t1__original_firstorder_90Percentile	0.007782	18.956
t1__original_gldm_SmallDependenceEmphasis	0.007576	22.329
t2__original_glcm_MCC	0.007449	6.673
t1__original_glszm_GrayLevelVariance	0.007376	1.724
t1__original_glrlm_LongRunLowGrayLevelEmphasis	0.006919	13.912
t1__original_shape_SurfaceVolumeRatio	0.006877	6.972
t1__original_glszm_SizeZoneNonUniformityNormalized	0.006842	29.458
t1__original_glrlm_RunEntropy	0.006812	9.006
t1__original_glrlm_RunLengthNonUniformity	0.006785	17.737
t2__original_gldm_DependenceNonUniformity	0.006665	8.174
t2__original_glszm_GrayLevelNonUniformity	0.006635	3.741
t2__original_glrlm_LongRunHighGrayLevelEmphasis	0.006550	11.101
t2__original_glrlm_RunEntropy	0.006544	11.548
t2__original_glszm_SizeZoneNonUniformity	0.006495	3.669
t2__original_glszm_SmallAreaHighGrayLevelEmphasis	0.006411	3.018
t1__original_shape_Flatness	0.006365	1.974
t2__original_shape_Flatness	0.006287	3.247
t2__original_glrlm_LongRunLowGrayLevelEmphasis	0.006169	0.305
t2__original_shape_Maximum2DDiameterSlice	0.006136	0.397
t2__original_shape_MinorAxisLength	0.006051	0.245
t2__original_glszm_SmallAreaLowGrayLevelEmphasis	0.005979	3.138
t2__original_glszm_ZonePercentage	0.005944	0.272
t2__original_shape_MeshVolume	0.005814	7.053
t2__original_glcm_Imc2	0.005786	4.381
t2__original_glszm_GrayLevelVariance	0.005768	5.711
t2__original_firstorder_Range	0.005762	1.909
t1__original_glszm_GrayLevelNonUniformity	0.005708	11.571
t1__original_glszm_SizeZoneNonUniformity	0.005668	3.834
t1__original_glszm_ZoneVariance	0.005537	12.221
t2__original_gldm_SmallDependenceHighGrayLevelEmphasis	0.005457	1.170
t2__original_firstorder_Maximum	0.005396	2.358
t1__original_shape_Maximum2DDiameterRow	0.005358	1.891
t1__original_shape_LeastAxisLength	0.005355	3.801
t1__original_gldm_DependenceNonUniformityNormalized	0.005305	26.571
t1__original_shape_Sphericity	0.005257	3.642
t1__original_firstorder_Variance	0.005139	5.456
t2__original_firstorder_Minimum	0.005130	8.578
t1__original_glrlm_GrayLevelNonUniformity	0.005105	5.136
t2__original_glcm_ClusterProminence	0.005058	1.960
t2__original_glrlm_LowGrayLevelRunEmphasis	0.005051	3.200
t2__original_firstorder_Skewness	0.005017	10.822
t1__original_glszm_SmallAreaLowGrayLevelEmphasis	0.004895	5.912
t1__original_shape_MinorAxisLength	0.004893	0.912
t2__original_glcm_ClusterShade	0.004890	2.109
t2__original_glszm_SmallAreaEmphasis	0.004850	0.032
t1__original_gldm_DependenceNonUniformity	0.004837	4.587
t2__original_shape_Maximum2DDiameterRow	0.004797	2.049
t2__original_gldm_LargeDependenceHighGrayLevelEmphasis	0.004762	6.205
t2__original_glszm_ZoneVariance	0.004732	4.776
t1__original_firstorder_Skewness	0.004694	8.766
t2__original_glszm_LowGrayLevelZoneEmphasis	0.004573	2.606
t2__original_glrlm_ShortRunHighGrayLevelEmphasis	0.004515	5.085
t2__original_shape_SurfaceArea	0.004438	3.006
t1__original_shape_Maximum2DDiameterColumn	0.004424	2.569
t2__original_glszm_ZoneEntropy	0.004386	0.387
t1__original_shape_MeshVolume	0.004344	4.337
t2__original_firstorder_10Percentile	0.004279	5.675
t2__original_glszm_SizeZoneNonUniformityNormalized	0.004274	0.132
t2__original_shape_MajorAxisLength	0.004268	1.128

t1__original_glszm_SmallAreaHighGrayLevelEmphasis	0.004255	1.220
t2__original_glcm_MaximumProbability	0.004253	3.764
t2__original_shape_Maximum3DDiameter	0.004172	1.096
t1__original_shape_Maximum2DDiameterSlice	0.004172	0.424
t2__original_glrlm_GrayLevelNonUniformity	0.004166	2.614
t2__original_shape_Maximum2DDiameterColumn	0.004146	1.138
t2__original_glszm_LargeAreaHighGrayLevelEmphasis	0.004139	7.043
t2__original_glrlm_RunLengthNonUniformityNormalized	0.004052	3.817
t1__original_shape_Elongation	0.004016	0.773
t2__original_shape_Elongation	0.003867	0.894
t2__original_glrlm_ShortRunLowGrayLevelEmphasis	0.003814	0.389
t1__original_glrlm_LongRunHighGrayLevelEmphasis	0.003810	13.321
t2__original_glrlm_ShortRunEmphasis	0.003784	2.166
t2__original_shape_LeastAxisLength	0.003735	4.424
t2__original_glrlm_GrayLevelVariance	0.003732	1.872
t1__original_glrlm_RunVariance	0.003338	6.942
t2__original_gldm_SmallDependenceLowGrayLevelEmphasis	0.003322	3.055
t1__original_shape_Maximum3DDiameter	0.003284	2.145
t2__original_gldm_SmallDependenceEmphasis	0.003277	2.620
t1__original_shape_MajorAxisLength	0.003268	2.986
t2__original_ngtdm_Strength	0.003169	1.997
t2__original_gldm_DependenceVariance	0.003128	2.495
t2__original_glszm_LargeAreaLowGrayLevelEmphasis	0.003118	0.208
t2__original_ngtdm_Busyness	0.002467	0.231
gender_male	0.000591	2.331

Table S10. Full revised available-metadata model comparison output

Model	n	Macro-AUC	Weighted AUC	Accuracy	Balanced accuracy	Macro-F1	Weighted-F1
T2+clinical Logistic regression	409	0.737	0.713	0.531	0.540	0.536	0.530
T2+clinical Support vector machine	409	0.690	0.661	0.553	0.548	0.551	0.552
T2+clinical Random forest	409	0.716	0.692	0.545	0.519	0.530	0.539
T2+clinical Gradient boosting	409	0.710	0.694	0.560	0.536	0.546	0.556
Age+sex clinical baseline	409	0.665	0.651	0.469	0.492	0.478	0.469
Paired T1/T2 complete-case RF	299	0.735	0.704	0.575	0.543	0.554	0.572

Table S11. Random forest comparator and sensitivity LOCO bootstrap confidence intervals

Analysis	n	Metric	Estimate	CI low	CI high
Primary T2+clinical RF LOCO	409	auc_macro_ovr	0.716	0.678	0.755
Primary T2+clinical RF LOCO	409	auc_weighted_ovr	0.692	0.649	0.738
Primary T2+clinical RF LOCO	409	accuracy	0.545	0.496	0.592
Primary T2+clinical RF LOCO	409	balanced_accuracy	0.519	0.469	0.568
Primary T2+clinical RF LOCO	409	macro_f1	0.530	0.481	0.577
Primary T2+clinical RF LOCO	409	weighted_f1	0.539	0.492	0.585
Primary T2+clinical RF LOCO	409	brier_multiclass	0.567	0.529	0.605
Sensitivity paired T1/T2 complete-case RF LOCO	299	auc_macro_ovr	0.735	0.691	0.777
Sensitivity paired T1/T2 complete-case RF LOCO	299	auc_weighted_ovr	0.704	0.657	0.751
Sensitivity paired T1/T2 complete-case RF LOCO	299	accuracy	0.575	0.520	0.632
Sensitivity paired T1/T2 complete-case RF LOCO	299	balanced_accuracy	0.543	0.486	0.596
Sensitivity paired T1/T2 complete-case RF LOCO	299	macro_f1	0.554	0.494	0.605
Sensitivity paired T1/T2 complete-case RF LOCO	299	weighted_f1	0.572	0.513	0.632
Sensitivity paired T1/T2 complete-case RF LOCO	299	brier_multiclass	0.543	0.497	0.594

Table S12. Random forest comparator centre-held-out metrics with bootstrap AUC confidence intervals

Analysis	Held-out centre	n test	Macro-AUC	AUC CI low	AUC CI high	Accuracy	Balanced accuracy	Macro-F1
Primary T2+clinical RF	EMC	70	0.825	0.742	0.891	0.686	0.647	0.653
Primary T2+clinical RF	IU	62	0.618	0.435	0.869	0.500	0.494	0.417
Primary T2+clinical RF	MCF	130	0.703	0.634	0.770	0.431	0.392	0.318

Primary T2+clinical RF	NYU	147	0.752	0.682	0.811	0.599	0.531	0.543
Sensitivity paired T1/T2 complete-case RF	EMC	70	0.804	0.720	0.884	0.643	0.630	0.631
Sensitivity paired T1/T2 complete-case RF	IU	62	0.595	0.378	0.766	0.532	0.417	0.374
Sensitivity paired T1/T2 complete-case RF	MCF	20	0.452	0.236	0.623	0.450	0.261	0.265
Sensitivity paired T1/T2 complete-case RF	NYU	147	0.747	0.681	0.808	0.578	0.523	0.534

Table S13. Radiomics rows by centre, modality and risk label

Center	Modality	Risk label	Rows
EMC	t1	0	34
EMC	t1	1	22
EMC	t1	2	14
EMC	t2	0	34
EMC	t2	1	22
EMC	t2	2	14
IU	t1	0	3
IU	t1	1	46
IU	t1	2	13
IU	t2	0	3
IU	t2	1	46
IU	t2	2	13
MCF	t1	0	25
MCF	t1	1	42
MCF	t1	2	63
MCF	t2	0	25
MCF	t2	1	42
MCF	t2	2	63
NYU	t1	0	48
NYU	t1	1	76
NYU	t1	2	23
NYU	t2	0	48
NYU	t2	1	76
NYU	t2	2	23

Supplementary code files

The following code/configuration files are supplied separately in the revision package: cystx_radiomics_params.yaml and cystx_extract_radiomics.py.

Supplementary Figure Legends

Figure S1. Full-cohort mask voxel distribution by centre and modality.

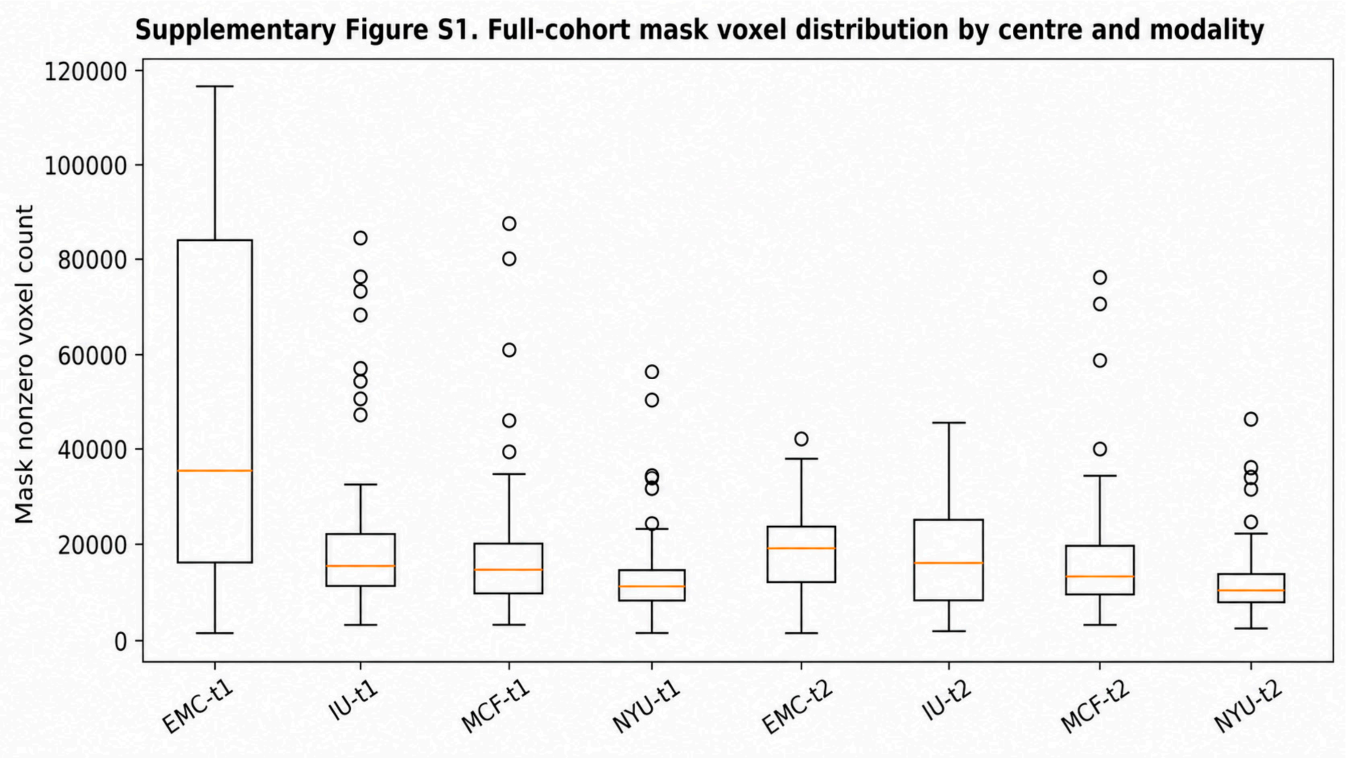

Boxplots showing non-zero mask voxel counts by centre and MRI modality after preprocessing. The figure supports the image-mask QC process and illustrates mask-volume heterogeneity across centres and modalities.

Figure S2. Distribution of pre-normalisation mean intensities by modality.

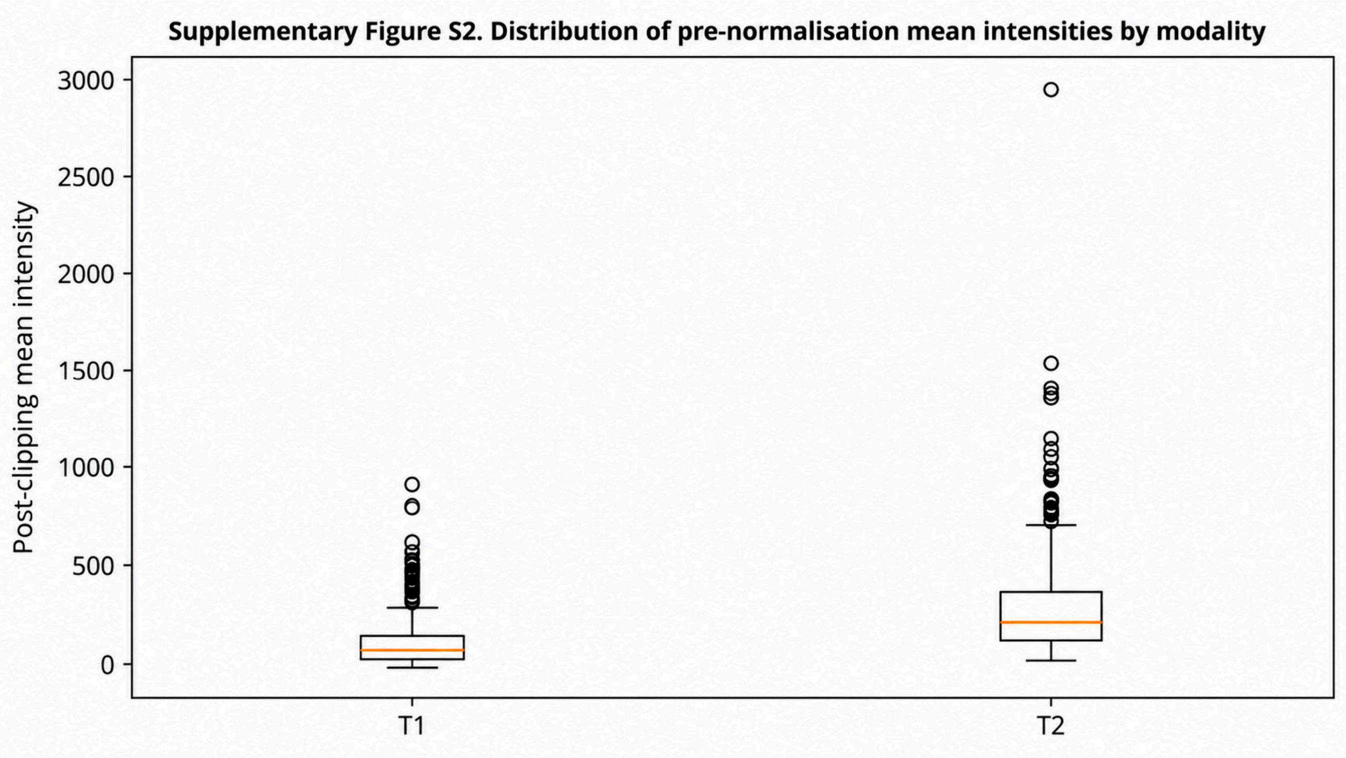

Boxplots showing pre-normalisation mean MRI intensities for T1- and T2-weighted images. The figure illustrates the need for intensity harmonisation before radiomics extraction.

Figure S3. Full-cohort image-mask QC summary.

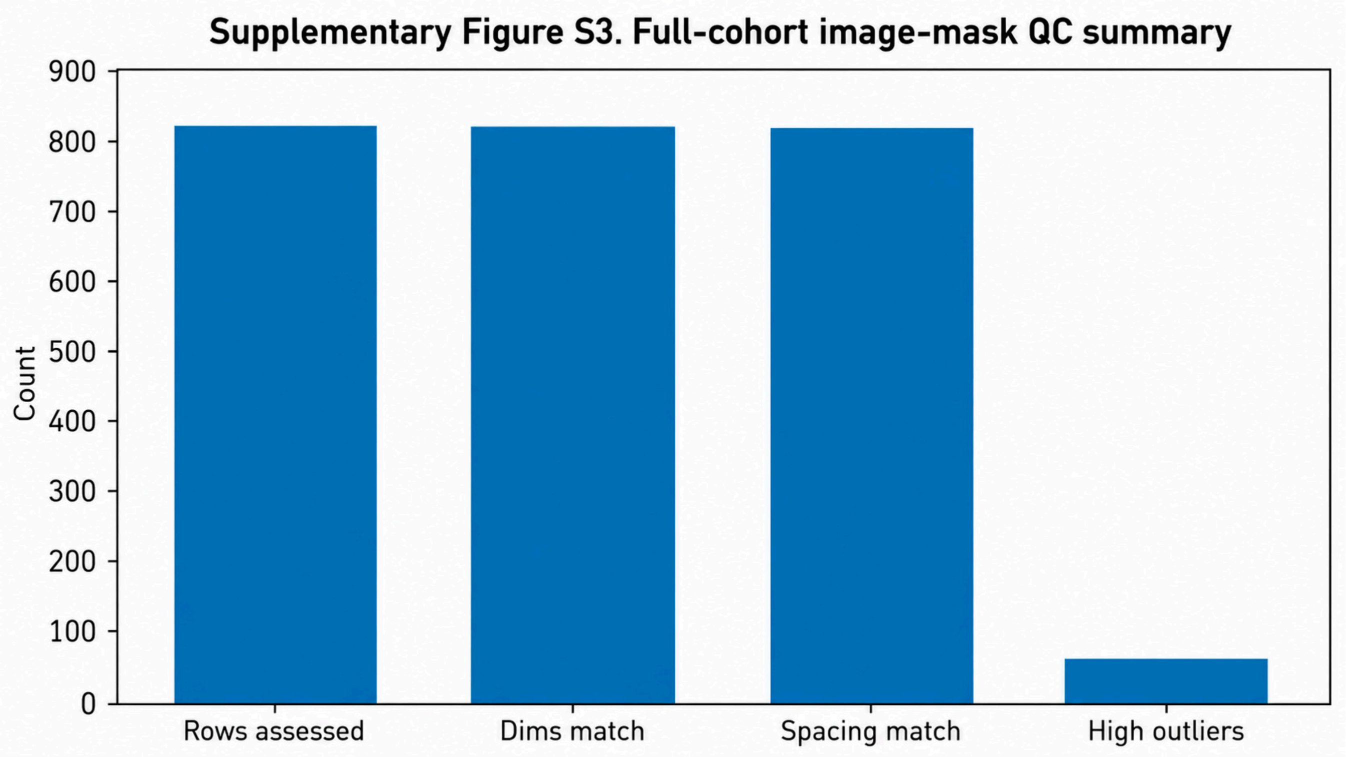

Summary of full-cohort image-mask QC results, including rows assessed, image-mask dimension matching, image-mask spacing matching and high-volume outlier flags.

Figure S4. Radiomics feature families in the raw extraction file.

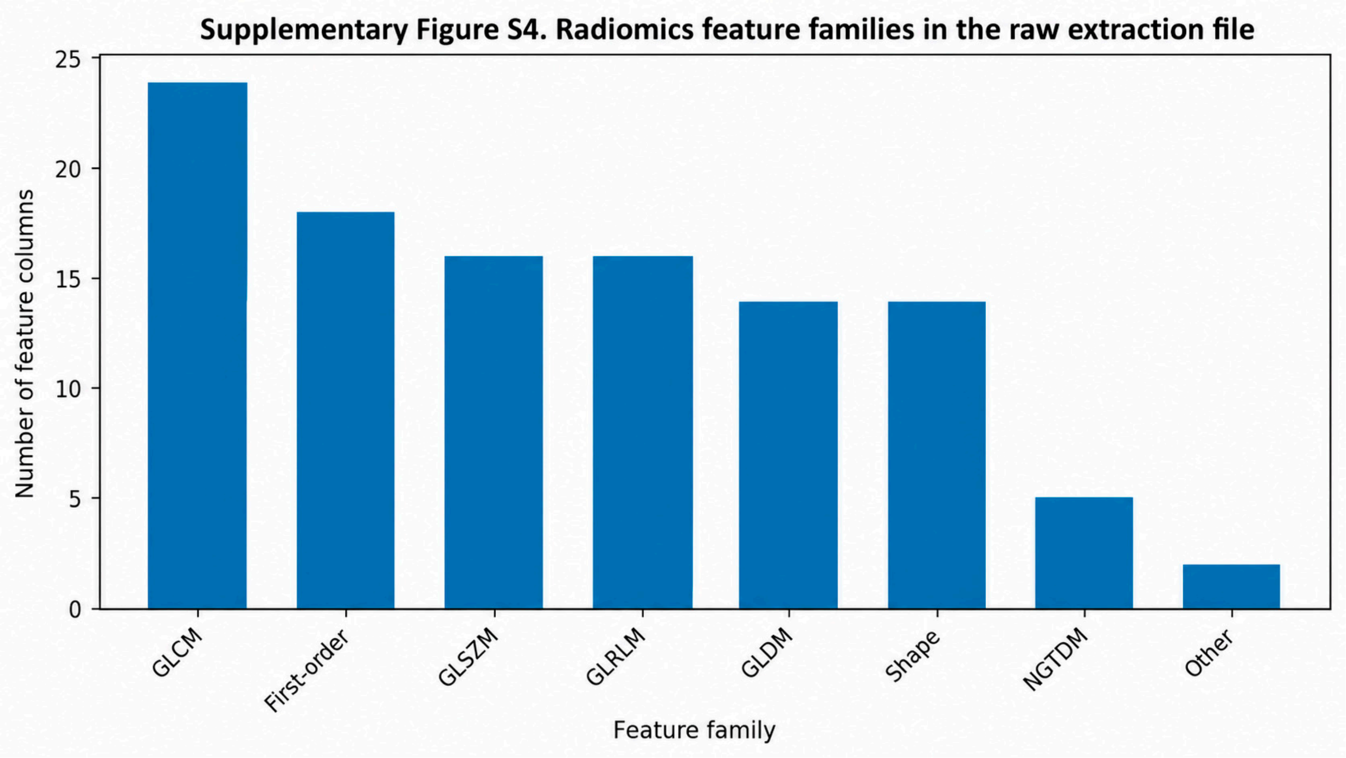

Bar chart summarising the radiomics feature families extracted from the raw PyRadiomics output, including GLCM, first-order, GLSZM, GLRLM, GLDM, shape and NGTDM features.
